# Retinal biomarkers for Alzheimer's disease in the prodromal and preclinical population ‐ The Deep and Frequent Phenotyping study

**DOI:** 10.1002/alz70856_101540

**Published:** 2025-12-25

**Authors:** Jamie Mitchell, Nowran Nasr, Eimear McLaughlin, Tony Thayanandan, Jennifer Lawson, Simon Lovestone, Vanessa Raymont, Adam Threlfall, Tom MacGillivray, Tunde Peto, Imre Lengyel, Lajos Csincsik

**Affiliations:** ^1^ Queen's University Belfast, Belfast, United Kingdom; ^2^ University of Oxford, Oxford, United Kingdom; ^3^ King's College London, Institute of Psychiatry and National Institute of Health Research (NIHR) Biomedical Research Centre for Mental Health, London, United Kingdom; ^4^ University of Oxford, Oxford, Oxfordshire, United Kingdom; ^5^ University of Edinburgh, Edinburgh, United Kingdom

## Abstract

**Background:**

Recent challenges for disease‐modifying therapies for Alzheimer's disease (AD) highlighted the need for biomarkers in the prodromal and preclinical stages. The Deep and Frequent Phenotyping (DFP) study aims to identify biomarkers for use in proof‐of‐concept trials. The DFP combines established AD markers with experimental approaches, including multimodal retinal imaging. Here, we report the baseline retinal vascular characteristics from ultra‐widefield retinal images and retinal thickness measurements from Optical Coherence Tomography (OCT).

**Method:**

Participants were recruited aged ≥60 years with prodromal AD and control participants in a 4:1 ratio. Retinal vascular parameters (RVPs) were extracted from OPTOS ultrawide‐field (UWF) images. Fractal dimension (FD), width gradient (WG), width intercept (WI), and tortuosity (TORT) were measured using the VAMPIRE software. OCT images were segmented with Heidelberg Eye Explorer (Heyex) to acquire the Retinal and Retinal Nerve Fibre Layer (RNFL) thickness. Associations between RVPs and AD risk factors (Family History of Dementia (FHD) and ApoE4 carrier status) were analysed in R, with results reported as medians and interquartile ranges.

**Result:**

FHD (*n* = 40) demonstrated significantly higher FD (1.13 [1.12, 1.15] vs 1.15 [1.13, 1.15], *p* <0.01) arterial WI (122μm [110, 132] vs 111μm [102, 129], *p* <0.05) alongside a lower arterial WG (‐3.28μm/mm [‐3.87, ‐2.74] vs ‐2.90μm/mm [‐3.28, ‐2.35], *p* <0.05) compared to those without FHD (*n* = 51). ApoE4 carriers (*n* = 11) exhibited reduced arteriolar (100μm [94, 115] vs. 115μm [105, 121], *p* <0.05) and venular (131μm [121, 142] vs. 152μm [136, 159], *p* <0.05) WI compared to non‐carriers (*n* = 80). No significant associations (*p* >0.05) were seen between TORT and FHD or ApoE4 status. FHD (*n* = 41) displayed a trend towards RNFL thickening in the Nasal (77μm [69, 87] vs 82μm [75, 90], *p* = 0.09) and inferotemporal (137μm [124, 148] vs 149μm [127, 158], *p* = 0.095) regions, compared to those without FHD (*n* = 48). No significant trends at baseline were observed between retinal thickness and FHD or ApoE4 status.

**Conclusion:**

These findings suggest that Retinal vascular parameters from OCT and UWF images may serve as non‐invasive biomarkers for AD risk, offering a scalable early detection and monitoring method. Future work will focus on longitudinal data to assess trends and their role in tracking disease progression.

